# Time-varying parameters of glycemic control and glycation in relation to arterial stiffness in patients with type 1 diabetes

**DOI:** 10.1186/s12933-022-01717-z

**Published:** 2022-12-09

**Authors:** Simon Helleputte, Patrick Calders, Arthur Rodenbach, Joke Marlier, Charlotte Verroken, Tine De Backer, Bruno Lapauw

**Affiliations:** 1 Faculty of Medicine and Health Sciences, Ghent University, Ghent University Hospital, Corneel Heymanslaan 10, 9000 Ghent, Belgium; 2 Fonds Wetenschappelijk Onderzoek (FWO) Vlaanderen, Ghent, Belgium; 3 Department of Cardiology, Ghent University Hospital, Ghent, Belgium; 4 Department of Endocrinology, Ghent University Hospital, Ghent, Belgium

**Keywords:** Type 1 diabetes mellitus, Arterial stiffness, Glycemic control, Glycation, HbA1c, Continuous glucose monitoring, Time in range

## Abstract

**Background:**

A substantial proportion of type 1 diabetes (T1D) patients free from known cardiovascular disease (CVD) show premature arterial stiffening, with age, blood pressure, and HbA1c—as gold standard of glycemic control—as main predictors. However, the relationship of arterial stiffness with other time-varying parameters of glycemic control and glycation has been far less explored. This study investigated the relationship of arterial stiffness with several short- and long-term parameters of glycemic control and glycation in patients with T1D, such as advanced glycation end-products (AGEs) and continuous glucose monitoring (CGM)-derived parameters.

**Methods:**

Cross-sectional study at a tertiary care centre including 54 patients with T1D free from known CVD. Arterial stiffness was assessed with carotid-femoral pulse wave velocity (cf-PWV). Current level and 10-year history of HbA1c were evaluated, and skin AGEs, urinary AGEs, and serum soluble AGE-receptor (sRAGE) concentrations. CGM for 7 days was used to determine time in range, time in hyper- and hypoglycemia, and glycemic variability.

**Results:**

Cf-PWV was associated with current HbA1c (r_s_ = + 0.28), mean 10-years HbA1c (r_s_ = + 0.36), skin AGEs (r_s_ = + 0.40) and the skin AGEs-to-sRAGE ratio (r_s_ = + 0.40), but not with urinary AGE or serum sRAGE concentrations; and not with any of the CGM-parameters. Multiple linear regression for cf-PWV showed that the model with the best fit included age, T1D duration, 24-h mean arterial pressure and mean 10-years HbA1c (adjusted R^2^ = 0.645, p < 0.001).

**Conclusions:**

Longer-term glycemic exposure as reflected by current and mean 10-years HbA1c is a key predictor of arterial stiffness in patients with T1D, while no relationship was found with any of the short-term CGM parameters. Our findings stress the importance of early and sustained good glycemic control to prevent premature CVD in patients with T1D and suggest that HbA1c should continue to be used in the risk assessment for diabetic complications. The role of skin glycation, as a biomarker for vascular aging, in the risk assessment for CVD is an interesting avenue for further research.

## Background

Arterial stiffness is a potential biomarker for complication risk in patients with type 1 diabetes (T1D), as its predictive role in the development of microvascular and cardiovascular disease (CVD) has been reported [1, 2]. We have recently demonstrated that about one quarter of patients with T1D free from known CVD show premature arterial stiffening [3]. Moreover, arterial stiffness strongly associated with the STENO risk score for future cardiovascular (CV) events and with CV imaging and function outcomes, illustrating the clinical relevance of arterial stiffness [3]. Considering its determinants, several patient- and disease-related factors have been investigated, with traditional risk factors age, blood pressure (BP) and glycated hemoglobin (HbA1c) being main predictors [3–6]. However, the relationship of arterial stiffness with other time-varying parameters of glycemic control and glycation in T1D, such as advanced glycation end-products (AGEs; long-term) and continuous glucose monitoring (CGM)-derived parameters (short-term), has been far less explored.

This is relevant, because as pointed out in a recent review in *The Lancet Diabetes & Endocrinology*, the landscape of glycaemia management based on HbA1c is changing [7]. The increasing use of CGM in T1D care has been a revolution [8], providing insight into different aspects of (shorter-term) glycemic control [9]. Nowadays, several parameters add value in clinical management [7], with time in range (TIR) increasingly being used next to glucose management indicator (GMI), time below and above range (TBR, TAR), and parameters of glycemic variability (GV) [9–11]. Importantly, TIR could provide complementary value (*i.e.* next to HbA1c) in predicting diabetic complications, and has been associated with microvascular complication risk in both T1D [12, 13] and type 2 diabetes (T2D) [14]. In T2D, there is also evidence on the association of TIR with CVD and mortality [15], while results from similar studies in T1D are not available yet. In addition, some studies have suggested an independent role of GV in developing microvascular complications in T1D [11, 16], although again the link with CVD is far less well-investigated.

Only few studies assessed the association of CGM-parameters with arterial stiffness in particular. Analyses of the Maastricht study showed significant associations of both TIR and GV with carotid-femoral pulse wave velocity (cf-PWV) in more than 800 participants of whom one-fourth had T2D [17], and a Japanese study in T2D showed that multiple CGM-metrics were associated with higher arterial stiffness [18]. However, to the best of our knowledge, no recent studies are available on the association between arterial stiffness and TIR or GV in T1D, and findings from T2D studies cannot be extrapolated considering the different pathophysiologic processes impacting vascular and glycemic health.

AGE levels, as can be measured in the skin by autofluorescence [19, 20] or in serum/plasma [20, 21], are higher in patients with T1D compared to non-diabetics [19–21], particularly in patients with complications [19, 21]. Until now, however, only one study has examined the relationship between AGEs and arterial stiffness in T1D patients, and found that skin AGEs but not serum AGEs were independently (*i.e.,* from HbA1c) associated with aortic stiffness [20].

This study aimed to evaluate the relationship of arterial stiffness with different time-varying parameters of glycemic control and glycation in patients with a T1D duration of at least 10 years but without known CVD.

## Methods

### Study design and subjects

Patients took part in this cross-sectional study [3] during 2019–2021 at our tertiary care centre if they met the following inclusion criteria: age > 18 years, T1D duration > 10 years and absence of known CVD (*i.e*., no history of angina pectoris, acute coronary syndrome, stroke, aortic disease, heart failure, symptomatic peripheral artery disease or any CV procedure or surgery) by consultation of patients’ electronic medical record as well as confirmed via patient reporting upon enrolment in the study. This study has been carried out in accordance with Ethical standards as mentioned in the Declaration of Helsinki for human experiments. The study was approved by the responsible Ethics Committee (EC-number: 2019/2090) and all patients provided written informed consent.

### Measurements

#### Patient and disease characteristics

Information on sex, age, smoking, T1D duration, insulin treatment, and body mass index (BMI) was collected. High-density lipoprotein (HDL)-cholesterol, total cholesterol (TC), and triglycerides (TGL) were measured on a fasting blood sample and low-density lipoprotein (LDL)-cholesterol was calculated (Friedewald equation). Ambulatory BP monitoring (ABPM) was performed, with brachial BP recorded for 24 h at the non-dominant arm (*Spacelabs Healthcare 90217A; Issaquah, WA, USA*). Systolic, diastolic and mean BP for daytime, night-time and 24 h were assessed. Data on serum creatinine, estimated glomerular filtration ratio (eGFR; CKD-EPI equation), and 24-h urine albumin-to-creatinine ratio (UACR) were evaluated; with diabetic kidney disease defined as albuminuria (UACR ≥ 30 mg/g creatinine or 30 mg/24 h), or use of a RAAS-inhibitor for albuminuria, or eGFR < 60 mL min^−1^ (1.73 m^2^)^−1^. Information on the presence of retinopathy and use of antihypertensive and lipid-lowering treatment was retrieved from patients’ electronic medical records.

#### Arterial stiffness: carotid-femoral pulse wave velocity (cf-PWV)

Arterial stiffness of the aortic segment was calculated as the travel distance of the pulse wave between the carotid and femoral artery, divided by the difference in transit time of the pulse wave between the heart (based on R-top identification on ECG) and these two locations [22, 23]. Measurements were performed with the SphygmoCor device (*AtCor Medical*^*®*^*, Sydney, Australia*) and according to consensus guidelines [23]. All patients were evaluated at the same time of day (8AM) to minimize influence of diurnal variation in blood vessel tone, after eight-hour overnight fasting and without intake of vasoactive medication, caffeine, tea, polyphenol-rich foods, alcohol, and nicotine and without performing strenuous exercise in the 24 h prior to testing. Measurements were only performed if glycemia was between 70 and 250 mg/dL. Measurements were performed in a quiet room after ten minutes of rest, with patients in supine position and not allowed to speak or sleep. Common carotid and femoral artery pulse waves were directly measured with applanation tonometry at the right side, with time delay in pulse wave arrival determined with the foot-to-foot method. The direct carotid-femoral distance was measured with an infantometer after precise determination of the arteries’ pulse location and 80% of the direct distance was used for cf-PWV calculation [22, 23]. A measurement was only accepted if the quality criteria indicated by the device were met; if the difference between two consecutive measurements was > 0.5 m/s, a third measurement was executed. The mean or median value of these two or three measurements, respectively, was used.

#### Long-term glycemic control and variability: HbA1c

Blood HbA1c was determined with high-performance liquid chromatography (HPLC) (*Automated Glycohemoglobin Analyzer HLC-723 G8; Tosoh*^*®*^* Bioscience Company, Tokyo, Japan*) to reflect patients’ current level of glycemic control, categorized as good (HbA1c ≤ 7.0% (≤ 53 mmol/mol), moderate [7.1–8.0% (54–64 mmol/mol)], poor [8.1–9.0% (65–75 mmol/mol)] or very poor [> 9% (> 75 mmol/mol)]. Mean and standard deviation (SD) of 10-years HbA1c history was collected to reflect patients’ long-term glycemic exposure and variability, respectively, provided that at least sixteen HbA1c values were available for the 10-year period and with a maximum of one year without HbA1c.

#### Measures of glycation

*Skin AGEs.* The accumulation of skin AGEs was evaluated non-invasively by measuring skin autofluorescence (SAF) using an AGE reader (*DiagnOptics BV, Groningen, The Netherlands*), as described elsewhere [24, 25]. The AGE value is the mean of three consecutive SAF measurements and expressed in arbitrary units (AU). Compared to age-based predicted values, patients are categorized into no, mildly, moderately, or strongly increased CV risk [25].

*Urinary AGEs.* UV-fluorescence spectroscopy was used for the detection and measurement of urinary autofluorescent (AF) AGEs [26, 27] on a 1 mL sample taken from the 24-h collection that had been stored at − 80 °C until centrifugation and analysis. AF measurements were performed by recording fluorescence spectra of the urine samples using a Flame miniature spectrometer (*FLAME-S-VIS–NIR-ES, 350–1000 nm, Ocean Optics, Dunedin, FL, USA*) equipped with a high-power LED light source (LLS *365 nm, Ocean Optics*) and reflection probe (*QR400-7-VIS-BX,* premium 400 µm, VIS/NIR, *Ocean Optics*). One mL urine was transferred into a quartz cuvette with a 1 cm path length. The reflection probe was positioned against the cuvette and a black background was used. The OceanView program (*Ocean Optics, Largo, FL, USA*) was set with an integration time of 10 ms and measurements were averaged over 128 scans. Using an excitation wavelength in the range of 340–407 nm, the excitation-emission spectra of urinary AGEs were recorded at a 407–670 nm emission range. After background correction, the fluorescence signal of each sample was measured. Normalized fluorescence spectra were prepared by dividing the relative fluorescence intensity at each wavelength by the (maximum) relative fluorescence intensity at the (corresponding) peak wavelength, *i.e.,* dividing the average light intensity emitted per nm for the 407–670 nm wavelength range (emission zone) by the average light intensity per nm over the 340–407 nm range (excitation peak), yielding the *emission:excitation-ratio* as a reflection of urinary AGE levels. As the urinary concentration of AGEs depends on the urine concentration, the relative fluorescence intensity (expressed in arbitrary units) was adjusted for the urinary creatinine concentration.

*Soluble receptor for AGEs (sRAGE).* Circulating total serum sRAGE concentrations were determined with a commercially available ELISA kit (DY1145, *Human RAGE Duoset ELISA, R&D Systems Europe, Ltd., Abingdon, UK)* on a 100 μL sample that had been stored at − 80 °C until analysis. The skin AGEs-to-sRAGE ratio (= [skin AGEs]/[sRAGE]) was calculated.

#### Short-term glycemic control and variability: continuous glucose monitoring (CGM)

A Dexcom G5 sensor *(Dexcom*^*®*^*; San Diego, California, USA*) was subcutaneously inserted in the lower abdomen measuring interstitial glucose concentration every 5 min for a 7-day period. Patients were instructed to rely on their usual glycemia measurement method, to maintain normal daily activities, and to not actively use the study CGM device or check the information depicted on the screen (except for calibration twice daily). Alarms for high or low glucose were disabled except the < 54 mg/dL alarm for ethical reasons. Data were extracted from the *Dexcom*^*®*^* Clarity* platform and processed with the web-based application *Glyculator 2.0*. The following CGM-parameters were collected: mean blood glucose (MBG), estimated HbA1c [% (mmol/mol)], TIR (70–180 mg/dL), TBR (total < 70 mg/dL and level 2 < 54 mg/dL), TAR (total > 180 mg/dL and level 2 > 250 mg/dL), area under the curve total (AUC_total_), AUC_hyper_ (> 180 mg/dL) and AUC_hypo_ (< 70 mg/dL) [28]. GV was evaluated by SD, coefficient of variation (CV% = SD/MBG), interquartile range (IQR), and mean amplitude of glycemic excursions (MAGE) for *within-day* GV; and SD between daily MBGs and mean of daily differences (MODD) for *between-day* GV [9, 11].

### Statistical analyses

All data were analyzed with SPSS Statistics software version 27.0 (*IBM Corp., Armonk, New York, USA*). Data were checked for normality with the Shapiro–Wilk test as well as visually by Q–Q plots and histograms, and shown as mean ± SD or median [P_25_–P_75_] depending on the distribution. Pearson (r) correlations were used to examine linear associations between normally distributed continuous variables, in any other case Spearman correlation (r_s_) was used. Multivariate linear regression (step-up approach) was used to investigate associations between one or more independent variables with the dependent variable of interest, *i.e.,* to evaluate predictors of cf-PWV. To in- or exclude predictor variables in the regression model, multicollinearity within the model was evaluated with the variance inflation factor (VIF)*,* with VIF > 4 denoting significant multicollinearity. Level of significance for all statistical tests was set at p < 0.05. An a priori sample size calculation for *F-test—Linear multiple regression fixed model* was performed, using G*Power software version 3.1.9.4. The regression model needs to contain at least the factors proven to be associated with arterial stiffness, being age, BP and HbA1c level [3, 5, 29]. Additionally, we estimated that the model should be adjusted for two or three other covariates depending on the results of the univariate associations. With an estimated effect size f^2^ supposed to be moderate (= 0.25), a maximum of five predictors in the model, a power of 0.80 and α-level at p < 0.05, this resulted in n = 48 patients that needed to be included. Therefore, we aimed to recruit at least 50 patients in this study.

## Results

### Patient characteristics

Fifty-four patients (n = 54; 32 male, 22 female) aged 46 ± 9.5 years (range: 26–68 years) and with long mean disease duration (27 ± 8.8 years) were included. All patient characteristics are shown in Table 1. Twenty-five patients (46.3%) were using statins or other lipid-lowering drugs. Sixteen patients (29.6%) were hypertensive on 24 h-ABPM, and twenty-one patients (38.9%) were using antihypertensive medication.

**Table 1:** Patient characteristics

Parameter		Mean ± SD /Median [P_25_–P_75_]	Subgroups	n (%)
T1D duration (years)		27 ± 8.8	10–19 years	8 (14.8)
			20–29 years	29 (53.7)
			≥ 30 years	17 (31.5)
Age at T1D onset (years)		19 ± 10.1	–	
BMI (kg/m^2^)		25.4 ± 3.88	< 25.0	27 (50.0)
			25.0–29.9	21 (38.9)
			≥ 30.0	6 (11.1)
Insulin administration method		–	MDI	39 (72.2)
			CSII	15 (27.8)
Total daily dose of insulin (TDD; units)		41 [34–75]	–	
Insulin sensitivity (= 1800/TDD) (mg/dL)		45 ± 16.3		
Routine point of care glucose measurement method		–	SMBG	3 (5.6)
			Flash CGM	40 (74.1)
			Real-time CGM	11 (20.4)
Smoking	–	–	Currently (yes)	4 (7.1)
			Previously (yes)	18 (33.3)
	Pack years	11 ± 7.7	–	
Microvascular complications		–	Diabetic kidney disease (yes)	18 (33.3)
		–	Retinopathy (yes)	24 (44.4)
Lipid profile (mg/dL)	TC	170 ± 26.5	–	
	HDL-C	59 ± 14.0		
	LDL-C	95 ± 21.1		
	TGL	68 [54.5–90.8]		
24-h ABPM (mmHg)	24-h SBP/DBP	119/73 ± 10.7/6.2	–	
	Daytime SBP/DBP	124/76 ± 11.8/6.6		
	Night-time SBP/DBP	109/64 ± 10.0/6.2		

*MDI* multiple daily injections, *CSII* continuous subcutaneous insulin infusion, *SMBG* self-monitoring of blood glucose with finger-prick, *ABPM* ambulatory BP monitoring

### Arterial stiffness: Carotid-femoral pulse wave velocity (cf-PWV)

Cf-PWV was measured in 50 patients (in two patients a reliable femoral pulse could not be obtained due to obesity; in two other patients the software failed to detect the R-wave on ECG due to presence of left bundle branch block). Median cf-PWV was 8.3 [6.8–10.1] m/s, ranging from 5.1 to 14.5 m/s.

### Univariate associations of cf-PWV with patient and disease characteristics

Cf-PWV showed moderate to good associations with traditional CV risk factors age (r_s_ = + 0.69, p < 0.001), T1D duration (r_s_ = + 0.41, p < 0.01), brachial office SBP (r_s_ = + 0.46, p < 0.001), and 24-h brachial SBP and MAP (r_s_ = + 0.52, r_s_ = + 0.45, p < 0.01). There were significant associations between cf-PWV and renal parameters serum creatinine (rs = + 0.36; p < 0.05), eGFR (rs = − 0.47, p < 0.001) and UACR (rs = + 0.39, p < 0.01). Cf-PWV was not significantly different between men and women (8.7 ± 2.20 m/s vs. 8.2 ± 2.20 m/s, respectively, p = 0.377), and was not associated with age at onset of T1D or with other metabolic markers BMI, waist circumference, total daily insulin dose or lipid profile parameters.

### Relationship between cf-PWV and parameters of glycemic control and glycation.

Parameters of glycemic control and glycation are shown in Table 2; and their mutual associations and with cf-PWV are listed in Table 3. The longer the period reflected by a parameter of glycemic control/glycation (current HbA1c < mean 10-years HbA1c < skin AGEs), the higher was the correlation coefficient of the association with cf-PWV (current HbA1c: r_s_ = + 0.28; mean 10-years HbA1c: r_s_ = + 0.36; skin AGEs: r_s_ = + 0.40). Cf-PWV was not associated with serum sRAGE concentrations or urinary AGEs, but did significantly associate with the skin AGEs-to-sRAGE ratio (r_s_ = + 0.40). Cf-PWV was not associated with HbA1c-variability (*i.e.* SD-HbA1c over 10 years).

**Table 2:** Parameters of glycemic control and glycation

Outcome parameter				Mean ± SD/ Median [P_25_–P_75_]		n (%)
Current HbA1c (% (mmol/mol))				7.8 ± 0.83 (62 ± 7.1)	≤ 7.0 (≤ 53)	10 (18.5)
					7.1—8.0 (54—64)	25 (46.3)
					8.1—9.0 (65—75)	16 (29.6)
					> 9.0 (> 75)	3 (5.6)
Mean 10-years HbA1c (% (mmol/mol))				7.7 ± 0.63 (61 ± 5.8)	–	
Skin AGEs (arbitrary units; AU)				2.4 ± 0.47	–	
CV risk interpretation based on skin AGEs (n = 53)				–	Not increased	7 (13.2)
					Mildly increased	15 (28.3)
					Moderately increased	21 (39.6)
					Strongly increased	10 (18.9)
Urinary AGEs (emission:excitation-ratio)				0.073 [0.047–0.115]	–	
Serum sRAGE (pg/mL)				908 [736–1172]	–	
Skin AGEs-to-sRAGE ratio				25.5 × 10^–4^ [18.61 × 10^–4^ − 32.17 × 10^–4^]		
CGM- parameters	TIR (%)			66 ± 12.9	–	
	TAR (%)		Total > 180 mg/dL	30 ± 14.2		
			Level 2 > 250 mg/dL	5.5 [1.8–9.4]		
	TBR (%)		Total < 70 mg/dL	3.6 [1.7–6.1]		
			Level 2 < 54 mg/dL	0.5 [0.08–1.43]		
	CV (%)			35 ± 6.1		

*AGEs* advanced glycation end-products, *sRAGE* soluble receptor for AGEs, *CGM* continuous glucose monitoring, *TIR* time in range, *TAR* time above range, *TBR* time below range, *CV* coefficient of variation

**Table 3:** Associations between the different parameters of glycemic control and glycation, and carotid-femoral pulse velocity (cf-PWV)

Parameter	Current HbA1c	Mean 10-years HbA1c	Skin AGEs	Urinary AGEs	sRAGEs	cf-PWV
Current HbA1c	–	–	–	–	–	**r**_**s**_ = + 0.28*
Mean 10-years HbA1c	r = + 0.71***	–	–	–	–	**r**_**s**_ = + 0.36*
Skin AGEs	NS	r = + 0.42**	–	–	–	**r**_**s**_ = + 0.40**
Urinary AGEs	NS	NS	**r**_**s**_ = + 0.30*	–	–	NS
sRAGE	NS	NS	NS	NS	–	NS
Skin AGE-to-sRAGE ratio (= [skin AGE]/[sRAGE])	NS	NS	**r**_**s**_ = + 0.61***	NS	**r**_**s**_** = **− 0.80***	**r**_**s**_ = + 0.40**

*AGEs* advanced glycation end-products, *sRAGE* soluble receptor for AGEs; Significant results are shown in bold,*p < 0.05; **p < 0.01; ***p < 0.001; NS: not significant (all NS p-values were above p = 0.183)

Bivariate models showed that skin AGEs predicted cf-PWV independent from current HbA1c and mean 10-years HbA1c (p < 0.01), while the abovementioned association of the skin AGEs-to-sRAGE ratio with cf-PWV was not independent from skin AGEs.

Concerning CGM-derived (short-term) glycemic control, cf-PWV was not associated with any of the CGM-parameters (TIR, TBR, TAR or GV parameters; all p-values > 0.26).

### Multiple linear regression for cf-PWV

**Fig. 1:**
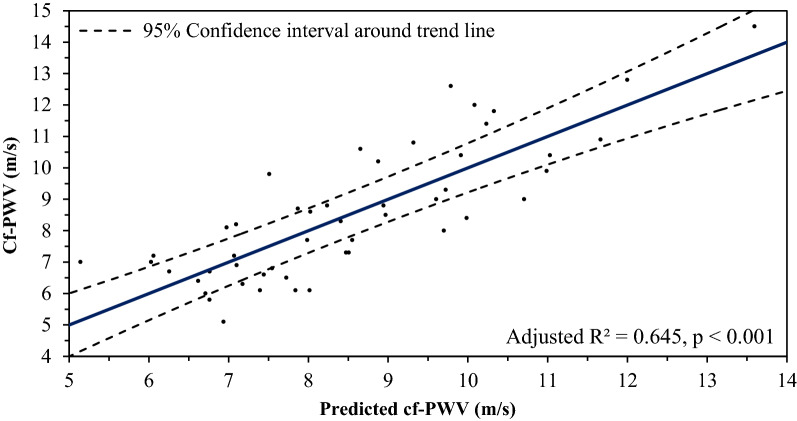
Multiple linear regression for predicting carotid-femoral pulse wave velocity (cf-PWV) with age, T1D duration, 24-h-MAP and mean 10-years HbA1c. (Regression equation: *Cf-PWV (m/s)* = − *10.457* + *0.085* × *Age (years)* + *0.129* × *T1D duration (years)* + *0.058* × *24-h-MAP (mmHg)* + *0.795* × *Mean 10-years HbA1c (%))*

The regression model (enter-, forward- and stepwise approach yielded the same results) that achieved the best fit for predicting cf-PWV included age, T1D duration, 24-h-MAP and mean 10-years HbA1c, explaining more than 60% of variance in cf-PWV (adjusted R^2^ = 0.645, p < 0.001) (Table 4, and Fig. 1). Note that: (a) current HbA1c and mean 10-years HbA1c could not simultaneously be included in a model due to collinearity, however also current HbA1c was a significant predictor in the multiple regression model with age, T1D duration and 24-h-MAP (adjusted R^2^ = 0.623, p < 0.001); (b) skin AGEs could not be included in a model simultaneously with age and mean 10-years HbA1c due to multicollinearity, and this was not overcome by standardizing the predictor variables; (c) neither in a 2nd model including [T1D duration + 24 h-MAP + mean 10-years HbA1c + skin AGEs] or in a 3rd model including [age + T1D duration + 24 h-MAP + skin AGEs], skin AGEs showed independent predictive value; (d) the model held when corrected for smoking status. There was no significant additional independent impact of adding the skin AGEs-to-sRAGE ratio, serum creatinine, eGFR or other outcome parameters to the abovementioned model.

**Table 4:** Multiple linear regression model for predicting carotid-femoral pulse wave velocity (cf-PWV) with age, T1D duration, 24-h-MAP and mean 10-years HbA1c

Model summary	Predictors: Age + T1D duration + 24-h MAP + Mean 10-years HbA1c
	Statistics: Adjusted R^2^ = 0.645, F (4,44) = 22.821, p < 0.001***
	Regression equation: *Cf-PWV (m/s)* = − *10.457* + *0.085* × *Age (years)* + *0.129* × *T1D duration (years)* + *0.058* × *24-h-MAP (mmHg)* + *0.795* × *Mean 10-years HbA1c (%)*
	Regression coefficients (Standardized Beta) and significance of individual predictors: Age: β = 0.370, p < 0.001*** T1D duration: β = 0.509, p < 0.001*** 24-h MAP: β = 0.206, p = 0.034* Mean 10-years HbA1c: β = 0.232, p = 0.025*

## Discussion

The present study found that in patients with T1D free from overt CVD, longer-term glycemic exposure as reflected by mean 10-years HbA1c is a key predictor of arterial stiffness. In contrast, although cf-PWV being associated with current HbA1c, no relationship was found with any of the short-term CGM-parameters. Our findings thereby (1) suggest that HbA1c should continue to be used in the risk assessment for diabetic complications, next to other glycemic control parameters such as TIR, complementing instead of competing each other; and (2) show the importance of early and sustained good glycemic control to prevent premature CVD in patients with T1D.

### Alternative measures of glycemic control: yes, we can go beyond but not without HbA1c.

Next to HbA1c, traditional CVD risk factors age and BP together with T1D duration [4–6, 30, 31] were the important correlates of arterial stiffness in our study population. A model including age, diabetes duration, 24-h-MAP and mean 10-years HbA1c explained more than 60% of variance in cf-PWV, which is a bit higher but in line with another publication with a similar though somewhat younger study population [5]. Our findings re-emphasize that early and sustained control of BP and HbA1c is crucial to slow vascular aging in T1D [32].

In recent clinical practice, besides HbA1c several CGM-derived (shorter-term) parameters became available, adding value in reflecting glycemic control and in all-day clinical management of T1D [33]. Although HbA1c remains a very good predictor for diabetic complications, it has been suggested that HbA1c might become less important in clinical management once more data demonstrate that TIR or other CGM-metrics add value in the risk assessment for diabetic complications [12]. It has even been argued that the good correlation between HbA1c and TIR permits the transition to TIR as preferred metric [12, 34].

In our study population, however, neither TIR or TAR nor parameters of GV were significantly associated with cf-PWV, despite that current HbA1c did so. A recent cross-sectional study conducting similar research in T1D neither found significant associations between TIR and carotid artery wall thickness or endothelial function [35]. Hence, our current findings are in favour of keeping HbA1c in the risk assessment for diabetic complications in T1D, complemented with instead of replaced by TIR, therefore we advocate for a multifactorial approach [36]. Indeed, it is expected that the landscape of glycemia management based on HbA1c is likely to change in favour of a more holistic approach considering all different aspects of dysglycemia [7]. Since it remains currently uncertain whether TIR contributes in the risk assessment for macrovascular complications in T1D, more prospective studies with uniform methodologies are needed to provide clear evidence on the added value of TIR and other CGM-metrics in estimating CVD risk.

Three points that could explain the lack of relationship between CGM-parameters and arterial stiffness should be noted. Firstly, inter-individual variation in glycation rate of HbA1c is an important explanatory variable in the relationship between HbA1c and TIR—and thus also in the association of TIR with arterial stiffness. Secondly, TIR was calculated over seven days whereas a sampling duration of 14 days has been suggested to reflect a patient’s level of glycemic control over the last three months more accurately [37]. Hence, it is possible that the 7-day period was not representative for a patient’s *average* level of glycemic control, thereby missing associations with the outcome. Thirdly, even though patients were asked to maintain normal daily activities and not to actively use the CGM device, it is not unlikely that they paid more attention to the device than they normally do. These three points are illustrated in our study by the finding that HbA1c was only moderately correlated with TIR (r = − 0.51). Lastly, it should be mentioned that glucose management and insulin delivery strategies have dramatically changed the past decade, with CGM use and newer insulin pumps leading to improvements in glycemic control. Patients who had shown poor control for years now show better glucose curves with substantially improved HbA1c (and TIR). Again, since vascular damage is primarily the result of *longer*-term exposure to hyperglycemia, this can explain why 10-years HbA1c and skin AGEs were more strongly (and independently) associated with cf-PWV than current HbA1c.

### Skin glycation as a marker for vascular aging?

Not unexpectedly, longer-term measures as mean 10-years HbA1c and skin AGEs showed stronger (and mutually independent) associations with arterial stiffness than current HbA1c. This again illustrates the importance of long-term glycemic exposure for vascular health as recently pointed out once more by DCCT analyses, advocating intensive insulin therapy aimed at lowering HbA1c as early and long as safely possible [38, 39]. The observed significant association between cf-PWV and skin AGEs confirms previous research in T1D where skin AGE accumulation was related to arterial stiffening independent from HbA1c [20], while serum AGEs were not. On the other hand, plasma AGEs were associated with incident CVD and mortality in a large 12-year follow-up study in T1D, and this independent from HbA1c and other CV risk factors, with the authors suggesting that AGEs constitute a specific target for treatment in these patients [40]. However, before systematic measurement of (skin or circulating) AGEs in clinical management of T1D might be considered, still more data are needed to evaluate if AGE measurements truly predict diabetes complications and especially CV risk independent from HbA1c [24] and from age, taking our findings into consideration.

Our study did not find associations of arterial stiffness with urinary AGEs or with sRAGE concentrations, while two prospective studies (9- and 12 years of follow-up) have shown independent predictive value of sRAGE levels for CVD and mortality [41, 42]. Notably, the skin AGEs-to-sRAGE ratio did associate with cf-PWV in our study. Two studies performed in the general population also found that skin AGEs and its ratio relative to sRAGE were most closely associated with arterial stiffness, with the authors suggesting these two to be better indicators of the current AGEs deposition status and more sensitive biomarkers of vascular aging than circulating AGEs [43, 44]. However, skin AGEs and its ratio to sRAGE were not included in the same regression model due to collinearity [43]. In our study, the skin AGEs-to-sRAGE ratio did not hold significance when skin AGEs was added to the model, so the question remains whether the skin AGEs-to-sRAGE ratio has independent predictive value for vascular complications, *i.e.,* independent from skin AGEs alone.

### Strengths and limitations

The strength of our study is that several possible correlates of arterial stiffness—assessed by the gold standard cf-PWV method—were evaluated in a well-described study population, such as various patient and disease characteristics, HbA1c history, AGE forms, and CGM-parameters. The limitation lies in its cross-sectional design, so that statements on causality cannot be inferred, and in its rather small sample size. The reported associations—or the lack thereof—now need to be further explored in follow-up studies, investigating the factors involved in the development and progression of arterial stiffness in T1D. Finally, although the added value of arterial stiffness is mainly pronounced in patients without previous CVD, our CVD-free study cohort does not reflect the entire T1D population and our findings cannot be extrapolated to patients with established CVD.

## Conclusions

The present study demonstrated that longer-term glycemic exposure as reflected by current and mean 10-years HbA1c is a key predictor of arterial stiffness in patients with T1D, while no relationship was found with any of the short-term CGM-parameters. Our findings stress the importance of early and sustained good glycemic control to prevent premature CVD in patients with T1D and suggest that HbA1c should continue to be used in the risk assessment for diabetic complications. The role of skin glycation, as a biomarker for vascular aging, in the risk assessment for CVD is an interesting avenue for further research.

## Data Availability

The data generated and/or analysed during the current study are not publicly available due to national legislation, but are available from the corresponding author on reasonable request.

## Abbreviations

AGE: Advanced glycation end products
BP: Blood pressure
cf-PWV: Carotid-femoral pulse wave velocity
CGM: Continuous glucose monitoring
CV: Cardiovascular
CVD: Cardiovascular disease
GV: Glycaemic variability
T1D: Type 1 diabetes
T2D: Type 2 diabetes
TIR: Time in range
